# Influenza A Virus M1 Protein Non-Specifically Deforms Charged Lipid Membranes and Specifically Interacts with the Raft Boundary

**DOI:** 10.3390/membranes13010076

**Published:** 2023-01-07

**Authors:** Anna S. Loshkareva, Marina M. Popova, Liudmila A. Shilova, Natalia V. Fedorova, Tatiana A. Timofeeva, Timur R. Galimzyanov, Petr I. Kuzmin, Denis G. Knyazev, Oleg V. Batishchev

**Affiliations:** 1 Laboratory of Bioelectrochemistry, Frumkin Institute of Physical Chemistry and Electrochemistry, Russian Academy of Sciences, 119071 Moscow, Russia; 2 Belozersky Institute of Physico-Chemical Biology, Lomonosov Moscow State University, 119991 Moscow, Russia; 3 Laboratory of Physiology of Viruses, D. I. Ivanovsky Institute of Virology, FSBI N. F. Gamaleya NRCEM, Ministry of Health of Russian Federation, 123098 Moscow, Russia; 4 Institute of Biophysics, Johannes Kepler University Linz, 4020 Linz, Austria

**Keywords:** influenza A virus, lipoprotein envelope, M1 matrix protein, hemagglutinin, viral budding, giant unilamellar vesicle (GUV), lipid raft, membrane deformation, amphipathic helices, fluorescent confocal microscopy

## Abstract

Topological rearrangements of biological membranes, such as fusion and fission, often require a sophisticated interplay between different proteins and cellular membranes. However, in the case of fusion proteins of enveloped viruses, even one molecule can execute membrane restructurings. Growing evidence indicates that matrix proteins of enveloped viruses can solely trigger the membrane bending required for another crucial step in virogenesis, the budding of progeny virions. For the case of the influenza A virus matrix protein M1, different studies report both in favor and against M1 being able to produce virus-like particles without other viral proteins. Here, we investigated the physicochemical mechanisms of M1 membrane activity on giant unilamellar vesicles of different lipid compositions using fluorescent confocal microscopy. We confirmed that M1 predominantly interacts electrostatically with the membrane, and its ability to deform the lipid bilayer is non-specific and typical for membrane-binding proteins and polypeptides. However, in the case of phase-separating membranes, M1 demonstrates a unique ability to induce macro-phase separation, probably due to the high affinity of M1’s amphipathic helices to the raft boundary. Thus, we suggest that M1 is tailored to deform charged membranes with a specific activity in the case of phase-separating membranes.

## 1. Introduction

Enveloped viruses present many pathogens, including coronaviruses, human immunodeficiency virus (HIV), Ebola virus, influenza virus, etc. Despite belonging to different families, many enveloped viruses share common structural features. Their genetic material is wrapped into two shells: an inner protein scaffold formed by matrix proteins and an outer lipid membrane inherited from a plasma membrane of the infected cell [1]. The ultimate stage of cell infection by enveloped viruses is a budding of progeny virions from the host plasma membrane [2]. Most of the similar cellular processes of membrane remodeling, including vesicle formation during in-cell protein traffic [3], exocytosis [4,5], and synaptic transmission [6], require the well-orchestrated interplay of different proteins between each other and with lipid membranes [5,6]. At the same time, viral protein machinery is rather minimalistic, providing a minimal number of proteins (sometimes only one) for each step of the viral lifecycle [7,8]. Many reports indicate that matrix proteins do not need other proteins to drive the budding process [9,10,11,12]. Matrix proteins are the most abundant and highly conserved among viral proteins [13,14]. Matrix proteins are typically multifunctional: (i) They maintain the integrity and the overall structure of the virion, thus protecting the viral genetic material; (ii) They enable the disintegration of the protein scaffold under certain conditions, thus facilitating the release of the genetic cargo into the infected cell; (iii) At the step of formation and budding of progeny viral particles, matrix proteins build a new envelope assembly with other viral proteins and the lipid membrane. Molecular mechanisms underlying these functions are still not fully understood, especially the role of protein–lipid interactions in the assembly and budding of new virions.

The spatial organization of the viral protein scaffold is either helical (such as with the influenza virus [15], vesicular stomatitis virus [16], measles virus [17], and the Newcastle disease virus [18]) or near-spherical when formed by 2D arrays of matrix proteins (such as with HIV [19,20]). Currently, a few matrix protein-driven budding mechanisms have been proposed: (i) Scaffolding of the lipid membrane due to intrinsic curvature of matrix proteins [21,22], which predominantly bind to the lipid membrane via hydrophobic interactions [23]; (ii) Condensation of lipids in contacting monolayer via electrostatic interactions resulting in relief of the excess area through the membrane bending [12,24]; (iii) Triggering the assembly of ordered membrane domains (rafts) with high boundary energy to initiate the bulging of the membrane [25,26]. Additional factors inducing membrane curvature could facilitate the budding process, e.g., palmitoylation of the influenza A virus’s spike protein, hemagglutinin (HA) [27].

Influenza A’s matrix protein M1 is a 27.8 kDa protein comprising a globular N-terminal part (the so-called NM-domain) and a flexible and partially disordered C-terminal domain [15,28,29]. Crystallographic data for the N-terminal domain for pH 7.0 and 4.0 show it as a dimer and only differ in the interaction interface between monomers [30]. These studies do not detect any inclination between protein monomers. Thus, M1 does not trigger budding via mechanism (i) because there is no intrinsic curvature of the protein oligomers, contrary to the matrix protein of the Newcastle disease virus [18,21]. Nevertheless, the M1 protein is able to form helical oligomers in a solution without other viral proteins and interactions with the lipid bilayer [29,31].

For most of the viral lifecycle, M1 interacts with lipid membranes, such as the plasma membrane of the infected cell or the lipid envelope of the virion. M1 binds the lipid membrane by its N-terminal part, with the C-terminal domain responsible for oligomerization and interactions with the ribonucleoprotein complex (RNP) [32,33]. M1 electrostatically interacts with anionic lipids of the inner leaflet of the plasma/viral membrane [34]. Anionic phosphatidylserine (PS) enhances the M1 oligomerization on the membrane [35,36], probably leading to the formation of membrane invaginations [37,38]. The presence of phosphatidylinositol 4,5-bisphosphate (PIP2) clusters M1 protein, possibly due to the high surface charge density [39]. Nevertheless, M1 weakly adsorbs to noncharged lipids [40,41] and can partially incorporate into the contacting monolayer, possibly by its amphipathic helices [42,43], which means that hydrophobic interactions are also involved in the protein–membrane binding. These interactions would explain why ordered nanodomains affect the M1–lipid interaction [38] and play a vital role in influenza infectivity [26]. Such interactions would decrease the energy of the hydrophobic mismatch between spike glycoproteins, HA, and neuraminidase (NA) apparently residing in the L_o_ domain [44] from one side and the surrounding L_d_ phase from another.

Pleomorphism of influenza virions might be another indication that M1 scaffold assembly is determined by an M1–lipid interaction. Indeed, depending on the strain and host [45], virions can take a variety of shapes, ranging from nearly spherical with a diameter of approximately 100 nm to micrometers-long filamentous with a close diameter.

The M1–lipid interaction is pH-dependent. Low pH triggers the disintegration of the protein scaffold, which is required for viral fusion with the endosomal membrane and RNP release [8,46,47]. Neutral pH enables the self-assembly of the M1 scaffold at the plasma membrane of the infected cell [48].

A well-known and convenient model of the cell plasma membrane is giant unilamellar vesicles (GUV) [49]. This model was applied several times to study the effect of matrix proteins of different viruses on the budding process [12,50,51]. Here, we used the GUV model and fluorescent confocal microscopy to investigate the role of the M1–lipid membrane interactions, the excess membrane area, and the phase state of the lipid bilayer in the budding of progeny virions. Based on the obtained results, we propose the mechanism of the M1-driven budding of virus-like particles.

## 2. Materials and Methods

### 2.1. Isolation of the M1 Protein

Isolation and purification of the M1 protein were performed as described in [52]. In brief, the influenza A virus strain PR/8/34 (H1N1) was propagated in 10-day-old embryonic chicken eggs with further purification by centrifugation through 20% (vol/vol) sucrose in STE buffer (100 mM NaCl, 10 mM Tris-HCl, and 1 mM EDTA at pH 7.4) at 21,000 rpm (90,000× *g*) for 90 min at 8 °C in the SW 27.1 rotor of a Beckman Spinco L5-75 centrifuge. Isolation of the M1 protein was performed by acid solubilization of the viral membrane with non-ionic detergent NP-40 (Igepal, Sigma, St. Louis, MO, USA) in 50 mM 2-(N-morpholino) ethanesulfonic acid (MES) and 100 mM NaCl buffer at pH 4.0. The obtained protein solution with a concentration of 0.1–0.2 mg/mL was dialyzed against the same buffer containing a Bio-Beads SM-2 adsorbent (Bio-Rad, Hercules, CA, USA) pretreated with methanol for 18 h at 4 °C. Dialyzed protein solution was concentrated using Microcon membranes (Microcon, Ultracel YM-10 regenerated cellulose, MWCO 10 000, Millipore, Burlington, MA, USA) at 10,000 rpm for 2 h at 4 °C up to a final concentration of 0.2–0.3 mg/mL. The purity of the protein samples was determined by SDS-PAGE gel electrophoresis and trypsin in-gel hydrolysis/MALDI-TOF mass spectrometry [47].

### 2.2. Preparation of Giant Unilamellar Vesicles (GUVs)

For the formation of GUVs, we used the following lipids in different ratios: 1,2-dioleoyl-sn-glycero-3-phosphocholine (DOPC); 1,2-dioleoyl-sn-glycero-3-phospho-L-serine (DOPS); bovine brain phosphatidylserine (bPS); 1,2-disteraoyl-sn-glycero-3-phospho-L-serine (DSPS); 1,2-dioleoyl-sn-glycero-3-phosphoethanolamine (DOPE); egg sphingomyelin (SM); cholesterol (Chol). All lipids were purchased from Avanti Polar Lipids (Alabaster, AL, USA) and were used without further purification. For the fluorescent microscopy, we used the following labeled lipids: 1,2-dioleoyl-sn-glycero-3-phosphoethanolamine-N-lissamine rhodamine B sulfonyl (Rho-PE) (Avanti Polar Lipids, Alabaster, AL, USA), for the liquid-disordered part of the membrane, and BODIPY^®^-monosialganglioside (BODIPY-GM1) for the liquid-ordered (raft) membrane phase.

GUVs were obtained using the electroformation technique, according to the protocol described in [53]. A lipid solution of 1 mg/mL in chloroform was deposed in the amount of 10 μL on two pre-cleaned platinum wires with a diameter of 1 mm and length of 5 cm and dried under the argon stream. Then, the wires were pre-heated to 55 °C and placed in a polypropylene chamber 3 mm apart. The chamber was filled with the swelling buffer, which contained 190 to 220 mM sucrose, 5 mM NaCl, 1 mM HEPES, pH 7.0, and was also pre-heated to 55 °C to obtain osmolarity equal to 100 mM NaCl and 50 mM MES buffer. The chamber was sealed with Parafilm and placed in a thermostat at 45 °C. A function generator was then connected to the wires, and a sinusoidal signal with incremental amplitude (from 50 to 700 mV) and 10 Hz frequency was applied for 3 h. The GUVs were then left to grow for 3 h and collected in polypropylene tubes. GUVs were stored at 4 °C no more than three days. The osmolarity of the GUVs suspension was checked before every measurement by the osmometer, and the osmolarity of the working buffer (100 mM NaCl and 50 mM MES, pH 7.0) was adjusted with glucose to satisfy isosmotic or hyperosmotic conditions.

### 2.3. Confocal Fluorescence Microscopy

**Figure 1:**
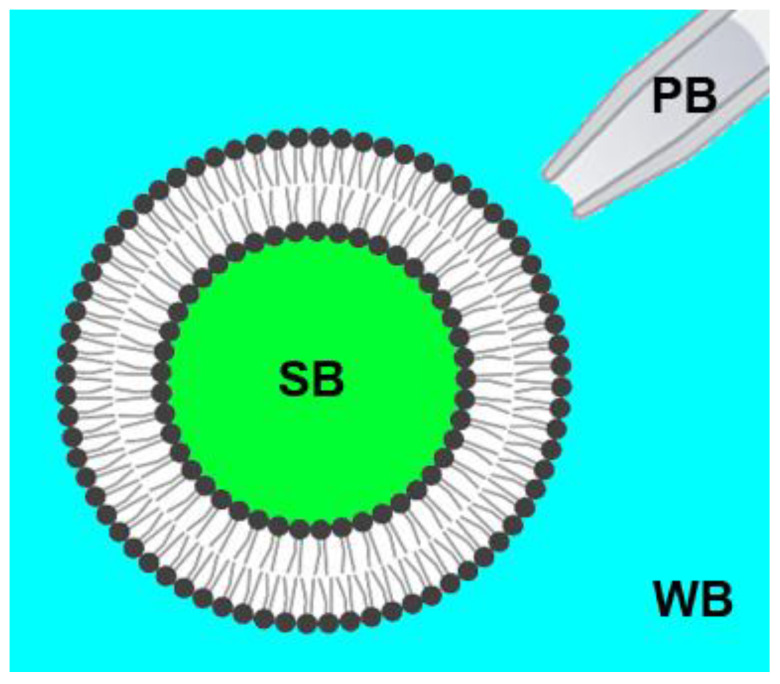
Scheme of the experiment. GUVs in the swelling buffer (SB) were added to the droplet of the working buffer (WB) and equilibrated for 15 min. M1 protein, as well as control proteins, BSA and PLL, were added in the pipette buffer (PB) to the selected GUV through a glass patch micropipette placed next to the selected GUV.

Imaging of GUVs was performed by a laser scanning microscope, LSM 510 META ConfoCor 3 (Carl Zeiss, Jena, Germany). For experiments, glass coverslips were covered with 1 mg/mL of bovine serum albumin (BSA) (Sigma, St. Louis, MO, USA) water solution for one hour. Then they were washed with deionized water and dried on air. This pretreatment is necessary to reduce GUVs flattening and rupture upon contact with the glass surface [54]. GUVs in the swelling buffer were added in a droplet of roughly 10 μL to the 100 μL droplet of the corresponding buffer and equilibrated for 15 min. After that, we focused on the vesicle of choice in its equatorial plane. GUVs with a diameter of 10 μm and greater were selected, and z-stack images were collected to ensure no protrusions or small vesicles inside the GUV. The procedure was repeated three times within 10 min before the protein addition. For each experiment, we analyzed 3–5 GUVs from three different preparations. M1 protein, as well as control protein BSA (Sigma, St. Louis, MO, USA) and polypeptide poly-L-lysine (PLL) (MW 70,000, Sigma, St. Louis, MO, USA), were added to the selected GUV through a glass patch micropipette placed next to the selected GUV. We used three types of experiments (isosmotic, dynamic hyperosmotic, and static hyperosmotic conditions), combining different relations between osmotic pressure (Π) of the swelling buffer (SB), working buffer (WB), and pipette buffer (PB) (Figure 1). For isosmotic experiments, the osmotic pressure of all buffers was equal: Π (PB) = Π (WB) = Π (SB). Dynamic hyperosmotic conditions were characterized by the high osmotic pressure of the pipette buffer (1 M NaCl, 50 mM MES, pH 7.0), while the rest of the buffers had similar osmolarity: Π (PB) > Π (WB) = Π (SB). Static hyperosmotic conditions were created by decreasing the osmolarity of the swelling buffer by 15% compared with the working and pipette buffers: Π (PB) = Π (WB) > Π (SB).

Control experiments for the effect of high concentrations of M1 and PLL on the GUV structure were performed in the epifluorescence mode to allow monitoring of overall changes in the vesicle.

The obtained images were processed using ZEN2009 (Carl Zeiss, Jena, Germany) and ImageJ (NIH, Bethesda, DC, USA) software.

## 3. Results

### 3.1. Interaction of the M1 Protein with GUVs in Isosmotic Conditions

It is commonly accepted that M1 binding to the cell membranes is predominantly electrostatic [32,34,55] and requires negatively charged lipids, e.g., phosphoserine (PS) [35,36,55]. Nevertheless, several works state that M1–membrane interactions are hydrophobic, including specific interactions with cholesterol [42,43,56]. Thus, to test the possible effects of M1, we prepared GUVs from the following lipid mixtures:

(1) DOPC:DOPE:Chol:Rho-PE = 59.99 mol%:30 mol%:10 mol%:0.01 mol% (herein referred to as Mixture 1);
(2) DOPC:DOPE:Chol:DOPS:Rho-PE = 50 mol%:24 mol%:10 mol%:15.99 mol%:0.01 mol% (herein referred to as Mixture 2).

**Figure 2:**
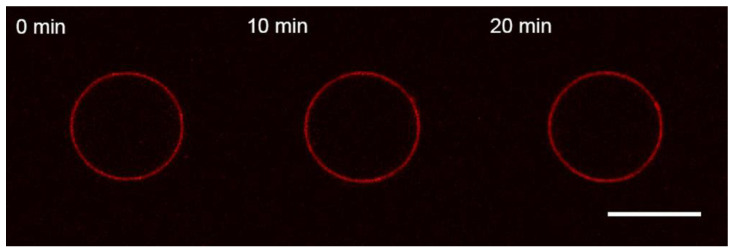
Monitoring the uncharged GUVs from the lipid Mixture 1 after 10 s perfusion with 1 μM M1 protein in the working buffer. The time count is synchronized with the perfusion start. The scale bar is 10 μm.

GUVs were added into the working buffer equilibrated by glucose to make isosmotic conditions with the swelling buffer inside GUVs. After selecting the GUV (see Materials and Methods for details), M1 was added via perfusion using a glass micropipette placed in the proximity of the GUV. The pipette buffer contained 1 μM of M1 in the working buffer solution. In the case of uncharged GUVs (Mixture 1), we did not observe any changes in the bilayer structure for over 20 min (Figure 2). This result is similar to the one for mono-component bilayers from DOPC for influenza C’s M1 [12] and the case of influenza A’s M1 adsorption on GUVs without charged lipids [37,57]. Control substances, BSA and PLL, also did not adsorb to the uncharged membranes (data not shown).

**Figure 3:**
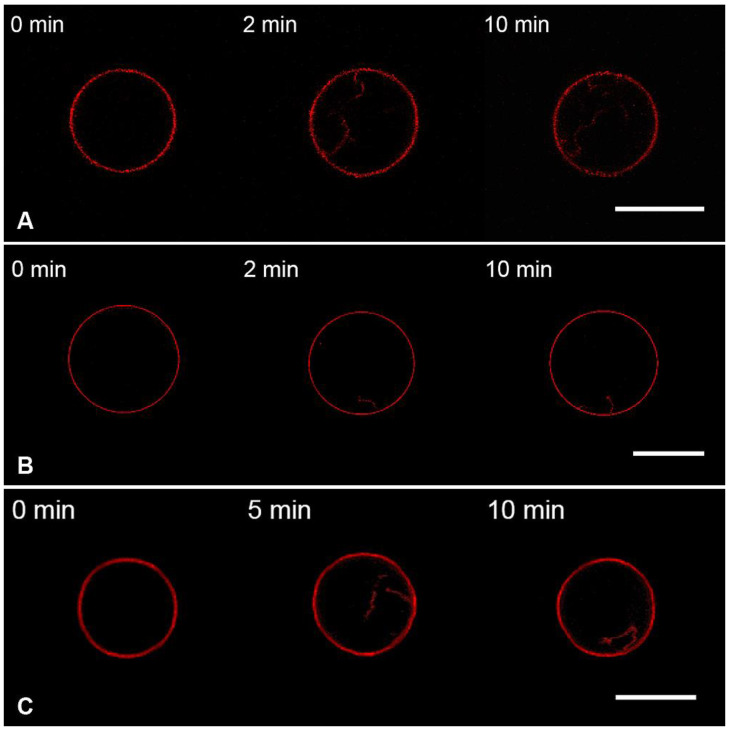
Monitoring the charged GUVs from the lipid Mixture 2 after 10 s perfusion with 1 μM of the M1 protein (**A**), BSA (**B**), or PLL (**C**) in the working buffer. Scale bars are 20 μm for (**A**,**B**) and 10 μm for (**C**).

In the case of charged GUVs (Mixture 2), the addition of M1 protein in the same conditions led to the formation of inward membrane protrusions (Figure 3A), in line with the obtained results for M1 of influenza C [12]. We compared the fluorescent intensities of the intact bilayer and protrusions using the approach introduced in [58]. For that, we made at least five cross-sectional profiles for each GUV with membrane filaments inside it, integrated fluorescent intensities for peaks corresponding to the GUV membrane and protrusions, and calculated the radii of membrane tubes (Figure S1 in Supplementary Materials). In this case, the estimated radius of the tubes was 61 ± 23 nm.

To check whether the growth of membrane protrusions was the effect specific to M1, we performed control experiments with BSA (Figure 3B) and poly-L-lysine, PLL (Figure 3C). These compounds should also adsorb on the charged GUVs by mere electrostatic interactions, without any preferences to exact lipids or membrane structure [59,60]. We performed the measurements in the same conditions for the M1 protein. We observed that both BSA and PLL adsorption yielded the growth of tubular protrusions inside the GUV. Radii of these protrusions were 94 ± 36 nm for BSA and 54 ± 19 nm for PLL.

Therefore, the formation of tubes from negatively charged GUVs is not the specific effect of M1 adsorption. Instead, electrostatic interaction between peptides and lipids may lead to the condensation of charged lipids beneath the protein molecule, resulting in the area imbalance of membrane monolayers. The observed tubular protrusions form as a result of the stress relief [61]. Because the protein adsorbs to the outer monolayer of the GUV, these protrusions are facing inwards [62].

Membrane tubulation is comparatively more pronounced at higher protein and anionic lipid concentrations. As it is shown in [37], significant deformations occur in the presence of 10 μM of M1 on the membranes containing more than 40% of negatively charged lipids. We used the same lipid composition (DOPC:DOPS:Chol:Rho-PE = 39.9 mol%:40 mol%:20 mol%:0.1 mol%) and indeed detected strong deformation of the GUV membrane upon perfusion with 10–11 μM of M1 (Figure S2A). However, the membrane of the same composition was already deformed at 2 μM PLL in the patch pipette (Figure S2B). We observed the formation of nearly spherical membrane invaginations, similar to those observed in [37] for the case of M1 adsorption. For higher concentrations of PLL, we detected rupture of GUVs. Therefore, membrane deformations upon M1 and PLL adsorption support the hypothesis of electrostatic condensation of the charged lipids as a budding driving force (budding mechanism (ii) in Introduction).

In terms of membrane elasticity, imbalance in monolayer areas is equivalent to the appearance of the spontaneous curvature in the monolayer contacting the protein. The radius of formed protrusions should depend on this curvature. Thus, for the given difference between the area of monolayers, the radius of the appearing membrane tubes should be the same. The lower the radius of protrusions, the larger imbalance it compensates. Therefore, a stronger protein-driven lipid condensation results in the membrane tubes with a lower radius.

Let us consider a GUV with a surface area $S_{0}=4\pi R_{0}^{2}$ and volume $V_{0}=4/3\pi R_{0}^{3}$, where *R*_0_ is the inner radius of the GUV. For the membrane thickness, *h*, the area imbalance between the outer and inner lipid monolayers will be $\Delta S_{0}=8\pi R_{0}h$. Adsorption of the protein on the outer membrane leaflet decreases this imbalance by the factor *α* < 1, resulting in the area of the outer monolayer *S_ex_* = *S*_0_(1 − *α*), where *S*_0_ = *S_in_* is the area of the inner monolayer. According to [62],

(1) $$S_{ex}-S_{in}+\Delta S_{0}=h\int^{\;}JdS,$$

where *J* is the membrane curvature.

If protein adsorption induces the formation of a cylindrical protrusion with the radius *r* and the total length *l*, then the GUV inner radius will reduce to *R* < *R*_0_ so that:

(2) $$4\pi R_{0}^{2}=4\pi R^{2}+2\pi rl.$$

Equation (1), in this case, gives

(3) $$8\pi R_{0}h-4\pi R_{0}^{2}\alpha =8\pi Rh-2\pi lh.$$

Expressing *α* through Equations (2) and (3) and taking into account that the radius of protrusions is much smaller than the radius of the GUV (*r* ≪ *R*_0_), we obtain:

(4) $$\alpha =h\left(\frac{R+R_{0}}{r}+2\right)\left(\frac{R_{0}-R}{R_{0}^{2}}\right)\approx h\frac{R_{0}^{2}-R^{2}}{R_{0}^{2}r}.$$

Re-arranging Equation (4), we obtain:

(5) $$r=h\frac{R_{0}^{2}-R^{2}}{R_{0}^{2}\alpha }=h\frac{S_{0}^{2}-S^{2}}{S_{0}^{2}\alpha }.$$

From Equation (5), one can see that the radius of protrusion is larger, with a bigger difference between *S*_0_ and *S*. Thus, to increase this radius, we should store the excess membrane area in its folds, which is the case when the vesicle is exposed to hypertonic conditions.

### 3.2. Interaction of the M1 Protein with GUVs in Hyperosmotic Conditions

To make the membrane-deforming effect more pronounced, we took advantage of the theoretical conclusion from Equation (5) and shrunk the vesicles prior to M1 adsorption. We used two modes to create the hyperosmotic conditions (i.e., shrinking the GUVs), dynamic and static. In the dynamic mode, swelling and working buffers were isosmotic, whereas the pipette buffer contained 1 M NaCl and 50 mM MES (pH 7.0). In the static mode, the swelling buffer had 15% lower osmolarity than the working buffer, and the latter was identical to the pipette buffer (see Materials and Methods and Figure 1). The static mode generates a moderate osmotic gradient across the bilayer of the whole GUV. In contrast, the dynamic mode locally creates a high osmotic gradient in the region of M1 adsorption.

In the static mode, we did not observe the formation of inward protrusions during the time of the experiment (30 min) for the uncharged GUVs (Mixture 1) upon perfusion with M1 (data not shown). Similarly, we observed no effect upon adding the control substances, BSA and PLL.

**Figure 4:**
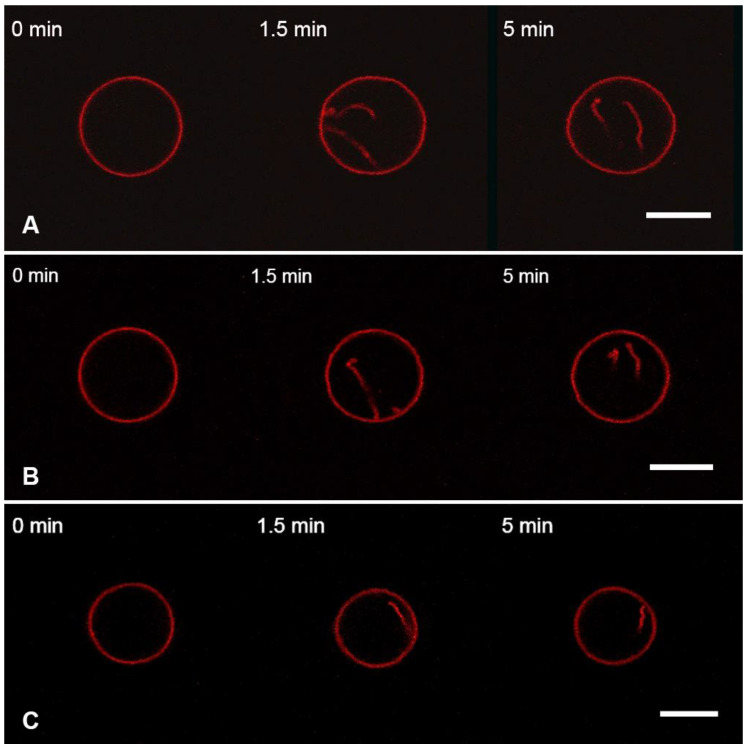
Monitoring the charged GUVs from the lipid Mixture 2 after 10 s perfusion with 1 μM of M1 (**A**), 1 μM of BSA, (**B**), or 1 μM of PLL (**C**) in the static hyperosmotic mode. The scale bar is 10 μm.

The addition of the M1 protein to the charged GUVs (Mixture 2) in static hyperosmotic conditions (Figure 4A) resulted in filamentous inward protrusions with a 150 ± 10 nm radius. This two-fold increase in the radius of protrusions, as compared to the isosmotic experiments, is in line with our theoretical predictions (Equation (5)).

Perfusion with the control substances BSA (Figure 4B) and PLL (Figure 4C) gave similar results. Perfusion with BSA resulted in protrusions with a radius of 127 ± 15 nm, and the radius was 158 ± 25 nm after perfusion with PLL.

**Figure 5:**
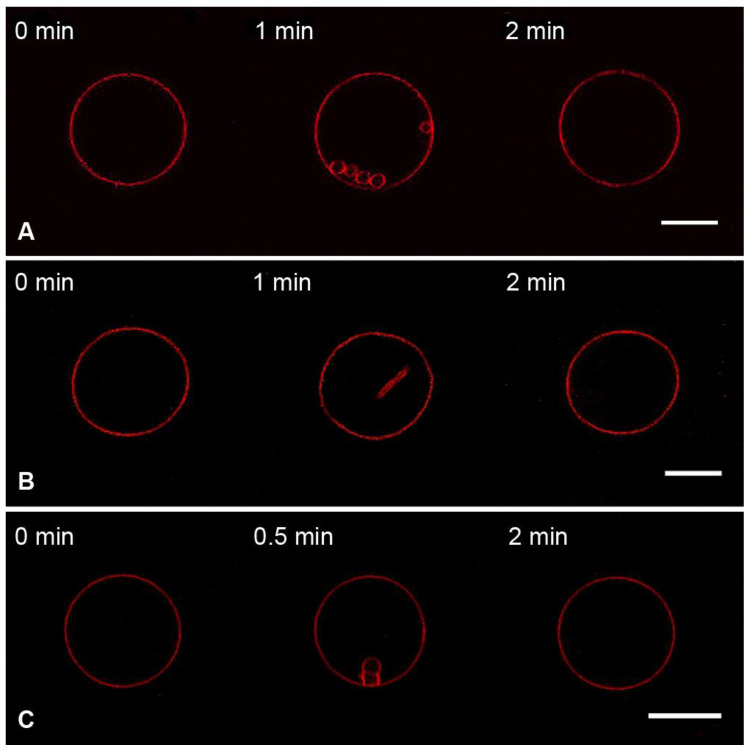
Monitoring the uncharged GUVs from the lipid Mixture 1 after 10 s perfusion with 1 μM of M1 (**A**), 1 μM of BSA (**B**), or 1 μM of PLL (**C**) in the dynamic hyperosmotic mode. The scale bar is 10 μm.

In the dynamic hyperosmotic mode, perfusion of uncharged GUVs (Mixture 1) with M1 yielded the formation of spherical particles inside GUVs that disappeared after perfusion stopped (Figure 5A). Similar effects were observed with BSA (Figure 5B) and PLL (Figure 5C).

The formation of progeny vesicles inside GUVs in hyperosmotic conditions (even in the absence of adsorbing proteins) has been known for decades. The proto-vesicles stay connected with the maternal membrane [63], which explains the transient character of the observed spherical structures (Figure 5). Our control experiments, where the perfusion solution did not contain protein, showed the same effect (Figure S3), confirming the osmotic nature of transient structures.

**Figure 6:**
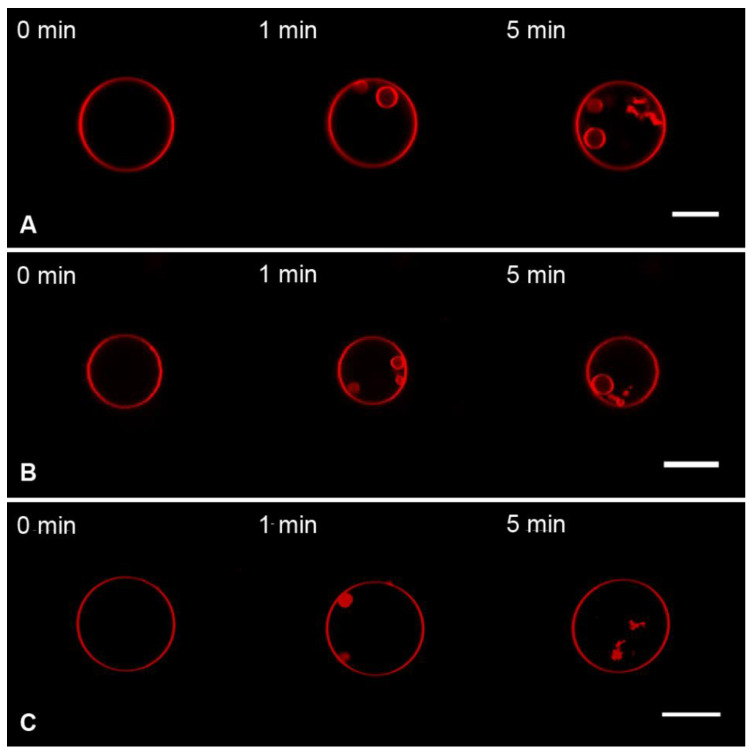
Monitoring the charged GUVs from the lipid Mixture 2 after 10 s perfusion with 1 μM of M1 (**A**), 1 μM of BSA (**B**), or 1 μM of PLL (**C**) in the dynamic hyperosmotic mode. The scale bar is 20 μm.

Perfusion of the charged GUVs (Mixture 2) with M1 in dynamic hyperosmotic mode led to the formation of filaments and spherical particles inside GUVs, which did not disappear after the perfusion was stopped (Figure 6A). Perfusion with BSA (Figure 6B) and PLL (Figure 6C) gave similar results.

**Figure 7:**
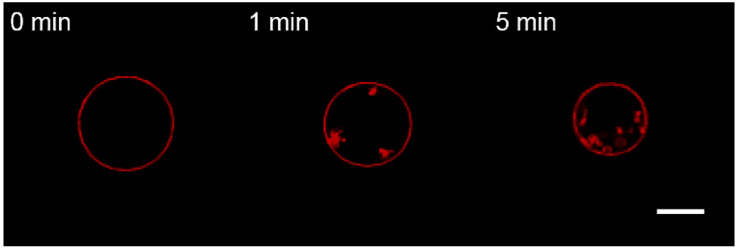
Monitoring the charged GUVs from the lipid Mixture 2 after 10 s perfusion with a protein-free solution in dynamic hyperosmotic mode. The scale bar is 20 μm.

Moreover, perfusion with the protein-free hyperosmotic solution also resulted in the formation of permanent inward structures (Figure 7). The exact mechanism of stabilization of such structures in charged membranes is unclear. Even though the dynamic hyperosmotic perfusion mode was used earlier to investigate the matrix protein–lipid interaction [54], it clearly poses severe artifacts and is unsuitable.

### 3.3. Interaction of the M1 Protein with the “Raft” GUVs

It is believed that the lipid envelope of the influenza virus has a liquid-ordered, or raft, nature. To mimic the viral membrane, we used the following GUV composition: bPS:Chol:SM:DOPC:BODIPY-GM1:Rho-PE = 20:33.3:33.3:13.3:0.01:0.01 (in mol.%) [26]. It includes both sphingomyelin and cholesterol for forming the raft (liquid-ordered, L_o_) phase and an extract of bovine brain phosphatidylserines, a mixture of PS lipids, approximately equal partitioning between the raft and non-raft phases [35]. Rho-PE served as a marker of the L_d_ (liquid-disordered, non-raft) phase, while BODIPY-GM1 was a marker of the L_o_ phase [64]. We used the isosmotic perfusion mode in experiments with the “raft” GUVs.

**Figure 8:**
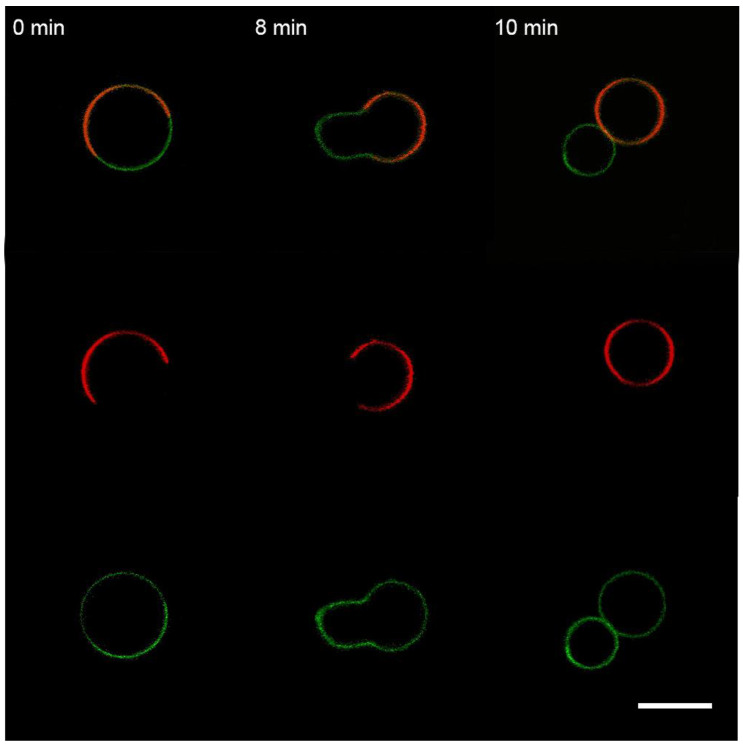
Monitoring the “raft” GUVs after 10 s perfusion with M1. The L_d_ marker Rho-PE is in the red channel, and the L_o_ marker BODIPY-GM1 is in the green channel. The scale bar is 20 μm.

We observed that perfusion with M1 causes a redistribution of L_o_ domains with the division of the GUV predominantly into L_o_ and L_d_ parts and, in some cases, with the division of the vesicle into two daughter vesicles (Figure 8).

**Figure 9:**
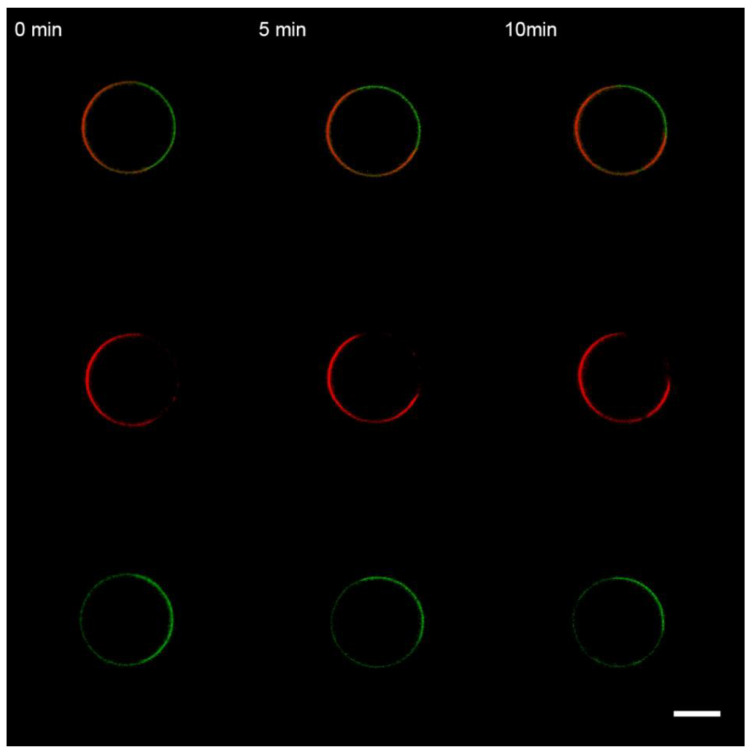
Monitoring the “raft” GUVs after 10 s perfusion with BSA. The color code is the same as in Figure 8. The scale bar is 20 μm.

**Figure 10:**
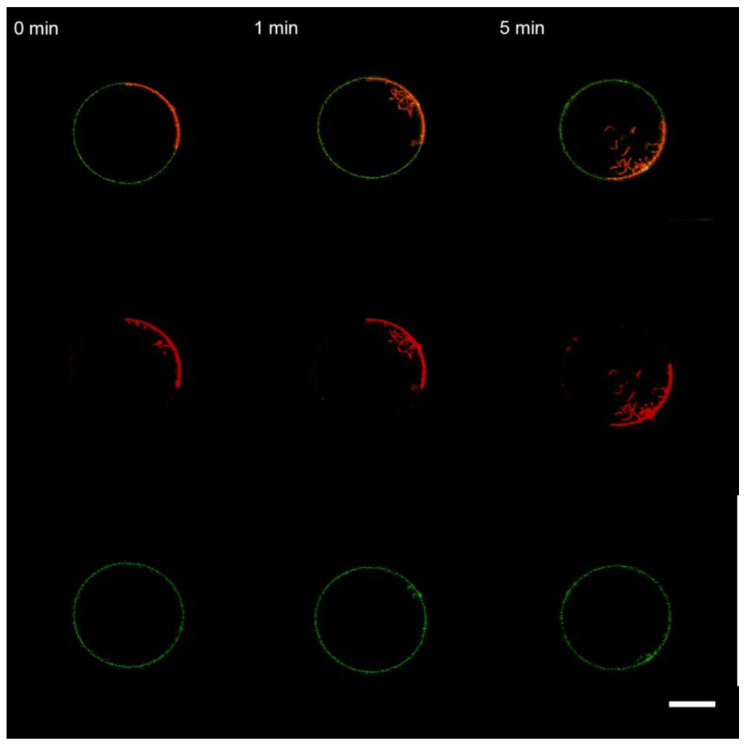
Monitoring the “raft” GUVs after 10 s perfusion with PLL. The color code is the same as in Figure 8. The scale bar is 20 μm.

In contrast to M1, perfusion with BSA did not lead to the division of GUVs on L_o_ and L_d_ parts (Figure 9). Perfusion with PLL resulted in the formation of filamentous protrusions predominantly from the L_d_ phase (Figure 10) but without division of the GUVs, as has been reported in [37].

Thus, for the “raft” GUVs, we found a difference in the membrane activity of M1, BSA, and PLL. To clarify the physicochemical mechanisms of protein-induced membrane deformations, we performed experiments with DOPS and DSPS instead of bPS. DOPS molecule has two unsaturated hydrocarbon chains and should mainly redistribute in the L_d_ phase, while DSPS with saturated lipid tails should predominantly partition into the L_o_ phase.

For the “raft” GUVs with DOPS instead of bPS (DOPS:Chol:SM:DOPC:BODIPY-GM1:Rho-PE = 20:33.3:33.3:13.3:0.01:0.01 in mol.%), perfusion with M1 protein yielded the deformation of the vesicle as a result of the separation of the GUV into L_o_ and L_d_ parts (Figure S4). In the case of BSA, we still observed the formation of filamentous and small spherical protrusions inside the GUV (Figure S5). At the same time, for PLL, we detected significant deformations of the GUV with the formation of polypeptide–lipid aggregates, resulting in a two-fold decrease in the vesicle size (Figure S6). The reason is that two unsaturated tails of DOPS possess much lower viscosity and bending rigidity than the mixture of saturated and unsaturated lipids in bPS. A similar effect was reported for the case of a high amount of M1 protein [37].

In the case of the “raft” GUVs with DSPS instead of bPS, the effects of perfusion with M1 (Figure S7) and PLL (Figure S8) were similar to the case of the charged GUVs from Mixture 2, with the formation of filaments and small spherical daughter vesicles. We noted that the L_o_ marker, BODIPY-GM1 (green channel in Figures S7 and S8), was uniformly distributed, whereas the L_d_ marker Rho-PE partitioned to the one half of GUV (red channel in Figures S7 and S8). This result supports the idea that more rigid and less mobile saturated lipids reduce the effect of protein adsorption and lipid condensation on GUV structure.

## 4. Discussion

The primary function of the matrix proteins is believed to be related to the assembly and budding of progeny virions from the plasma membrane of infected cells [65]. Several studies suggest that M1 can deform lipid membranes [37] or even produce virus-like particles in case of over-expression of the protein [66]. The ability to produce filamentous membrane protrusions upon adsorption is also detected for the matrix protein from the influenza C virus [12]. To clarify the physicochemical mechanisms of M1-membrane binding, we used GUVs with different lipid compositions (charged, uncharged, and raft-forming). We proved that M1 is able to deform GUVs containing negatively charged lipids, such as bPS (Figure 3A) or DOPS (Figure S2A), and did not bind to uncharged vesicles (Figure 2). Adsorption of the protein yielded the formation of filamentous protrusions inside the GUV. The same effect was observed for control substances, BSA and PLL. The formation of protein-free membrane filaments upon adsorption has been reported for other peripheral proteins, which produce an area imbalance between membrane monolayers due to lipid condensation [24,62]. On the other hand, for the matrix protein of the Newcastle disease virus [51], it is suggested that this protein alone can deform lipid membranes via the formation of 2D protein arrays in a solution [18], enforcing their geometry on the membrane [21]. The M1 protein is able to assemble into helical structures in a solution but without any pronounced rigidity or inclination angle between monomers [29]. However, in work [37], the authors show that M1 deforms the membrane to a great extent and forms a dense layer on the membrane. Therefore, we repeated these experiments under the same conditions (40 mol.% of DOPS in GUVs and 10 μM of M1) and found that, in this case, we also observed significant changes in the GUV structure (see Figure S2A). Control experiments with PLL (Figure S2B) manifested a greater restructuring effect of this polypeptide at a much lower concentration of 2 μM. Thus, we hypothesize that the membrane deformations resulted from osmotic or crowding effects of poly-charged M1 or PLL, which was also reported for other peripheral proteins [67,68,69] and even high salt concentrations [63]. That is why M1-induced membrane budding in cells has been reported for the case of an over-expressing vaccinia virus-driven system [10] and not for influenza A virus-infected cells [70]. Nevertheless, such concentration-dependent non-specific mechanisms of membrane bending may be the feature of the M1 protein. Recent studies of M1 co-clustering with PIP2 also suggest the primary role of electrostatics in M1-lipid binding. That is why we refrained from the fluorescent labeling of the protein with NHS-ester fluorophores because it would decrease the amount of free primary amines supposedly involved in M1’s interaction with the lipid bilayer. In the present study, we used an M1 protein directly purified from influenza A virions.

According to Equation (5), a protein’s ability to produce membrane filaments or daughter vesicles depends on the amount of excess area stored in membrane folds. Therefore, we performed experiments with the addition of M1 and control substances in hyperosmotic conditions, either with a small static osmotic gradient (static hyperosmotic conditions) or with a high dynamic osmotic gradient (dynamic hyperosmotic conditions). In the first case, we observed an increase in the radius of formed membrane filaments (Figure 4), while in the latter case, we detected the formation of spherical daughter vesicles (Figure 6) that proved our hypothesis about the origin of membrane curvature by mere lipid condensing effects of peripheral proteins adsorbing at the membrane.

One more option for the protein to produce bulging of the lipid bilayer is its possible influence on rafts [71]. Any substance acting on the line tension of the raft boundary [64,72] can produce the bowing of the membrane [25]. Here, we performed experiments with raft-forming GUVs and different types of anionic lipids, which should modulate the association of M1 with raft and non-raft phases (bPS), only non-raft phase (DOPS), and predominantly raft phase (DSPS). We observed that, in the case of bPS-containing raft GUVs, the addition of the M1 protein led to the division of the vesicle into a raft and a non-raft one (Figure 8). This is aligned with the observed widening of the electron density profile of the membrane with M1, as detected by SAXS in [38], due to the change of the initial spherical shape of the vesicle to a more prolate dumbbell one. BSA and PLL did not exhibit such behavior (Figure 9 and Figure 10). Changing bPS to DOPS increased the activity of PLL in condensing the charged lipids (Figure S6), while M1 was still able to bulge the raft phase, even though it was to a lesser extent (Figure S4). For DSPS, we observed only minor changes in membrane structure with the formation of filamentous protrusions (Figures S7 and S8). This could result from a much more rigid structure of the raft membrane [73], preventing its bending by proteins.

Thus, only the raft-forming GUVs reacted differently to M1 and other tested substances. In fact, the ability of the matrix protein to divide vesicles by lipid phases indicates the possible presence of amphipathic helices in its structure, as has been predicted earlier [43]. These helices would induce M1 activity on the line tension of the raft boundary [72] and lead to membrane bulging. Since the formation of the raft phase could result from the wetting of proteins by lipids [74], our observations indicate a new possibility of M1 activity on the stage of viral budding. Several studies indicate the possible involvement of cytoplasmic tails of HA and NA in the M1–membrane association [27,38,75], but the possible sites of such interactions are still unknown. Here we suggest another possibility: HA and NA may produce lipid rafts by their hydrophobic transmembrane domains, which, in turn, accumulate M1 and activates subsequent membrane bulging and budding by the line’s tension-driven mechanism. Indications that the M2 channel locates at the raft boundary [76] and interacts with M1 to stimulate budding fully support our idea.

## Data Availability

Data will be available upon reasonable request.

## Supplementary Materials

The following supporting information can be downloaded at: https://www.mdpi.com/article/10.3390/membranes13010076/s1, Figure S1: Fluorescent intensity profile across the charged GUV from the lipid mixture 2 after 10 s of perfusion with 1 μM of the M1 protein; Figure S2. Monitoring the charged GUVs from the mixture DOPC:DOPS:Chol:Rho-PE = 39.9 mol%:40 mol%:20 mol%:0.1 mol% after 10 s of perfusion with (A) 10 μM of M1; (B) 2 μM PLL in the isosmotic mode; Figure S3. Monitoring the uncharged GUVs from the mixture 1 after 10 s of perfusion with the protein-free hyperosmotic solution (1 M NaCl, 50 mM MES, pH 7.0); Figure S4. Perfusion with M1 of “raft” GUVs with DOPS instead of bPS (DOPS:Chol:SM:DOPC:BODIPY-GM1:Rho-PE = 20:33.3:33.3:13.3:0.01:0.01 (mol%)); Figure S5. The same as Figure S4, but for perfusion with BSA; Figure S6. The same as Figure S4, but for perfusion with PLL; Figure S7. Perfusion with M1 of “raft” GUVs with DSPS instead of bPS (DSPS:Chol:SM:DOPC:BODIPY-GM1:Rho-PE = 20:33.3:33.3:13.3:0.01:0.01 (mol.%)); Figure S8. The same as Figure S7, but for perfusion with PLL.
