# Supplementary material for: Influenza A Virus M1 Protein Non-Specifically Deforms Charged Lipid Membranes and Specifically Interacts with the Raft Boundary
Source: Membranes (Basel). 2023 Jan 7;13(1):76. doi: 10.3390/membranes13010076 (PMC9864314; doi:10.3390/membranes13010076)
Supplement: Supplementary file 1 [file membranes-13-00076-s001.zip › membranes-2086649-supplementary.pdf]

SUPPORTING INFORMATION

Influenza A Virus M1 Protein Non-Specifically Deforms Charged Lipid Membranes and Specifically Interacts with the Raft Boundary

Anna S. Loshkareva [1], Marina M. Popova [1], Liudmila A. Shilova [1], Natalia V. Fedorova [2], Tatiana A. Timofeeva [3], Timur R. Galimzyanov [1], Petr I. Kuzmin [1], Denis G. Knyazev [4,*] and Oleg V. Batishchev [1,*]

1 Laboratory of Bioelectrochemistry, Frumkin Institute of Physical Chemistry and Electrochemistry, Russian Academy of Sciences, 119071 Moscow, Russia
2 Belozersky Institute of Physico-Chemical Biology, Lomonosov Moscow State University, 119991 Moscow, Russia
3 Laboratory of Physiology of Viruses, D. I. Ivanovsky Institute of Virology, FSBI N. F. Gamaleya NRCEM, Ministry of Health of Russian Federation, 123098 Moscow, Russia
4 Institute of Biophysics, Johannes Kepler University Linz, 4020 Linz, Austria
* Correspondence: olegbati@gmail.com (O.V.B.); denis.knyazev@jku.at (D.G.K.)

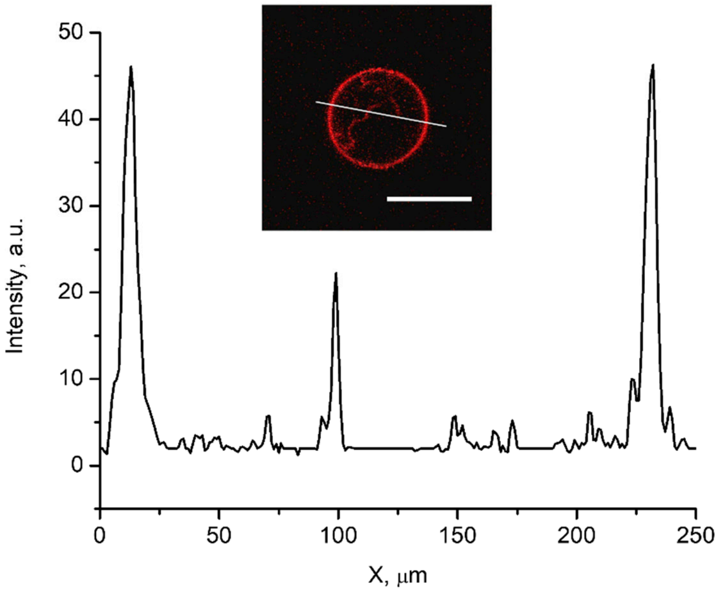

Figure S1. Fluorescent intensity profile across the charged GUV from the lipid mixture 2 after 10 seconds of perfusion with 1 μM of the M1 protein. X scale is set along the profile (white line in the inset. Scale bar in inset is 20 μm.

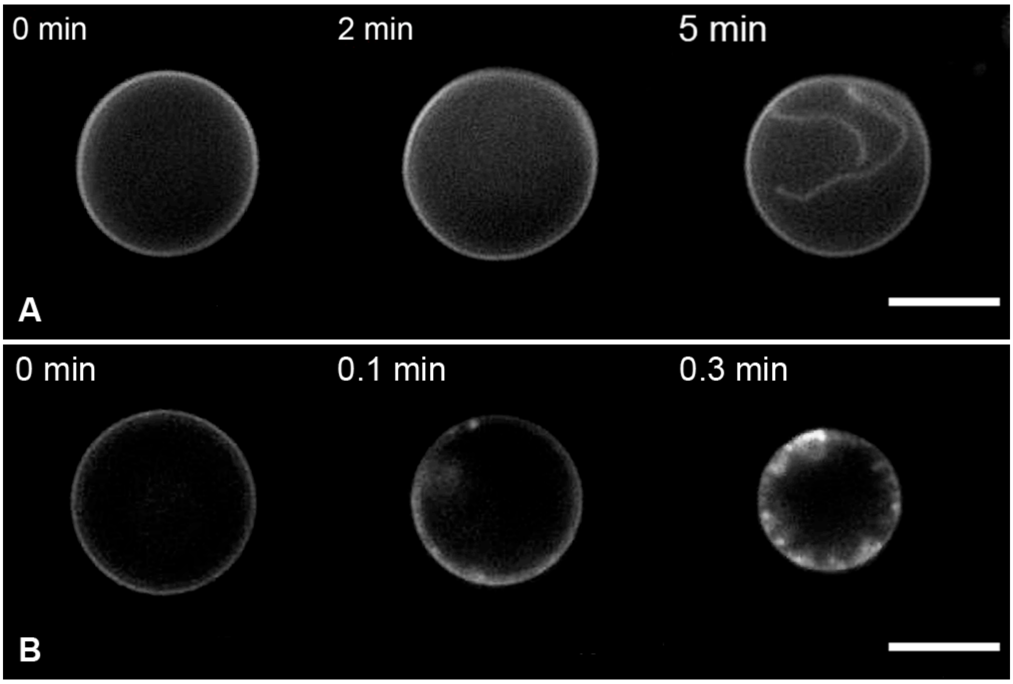

Figure S2. Monitoring the charged GUVs from the mixture DOPC:DOPS:Chol:Rho-PE = 39.9 mol%:40 mol%:20 mol%:0.1 mol% after 10 seconds of perfusion with (A) 10 μM of M1; (B) 2 μM PLL in the isosmotic mode. The scale bar is 10 μm.

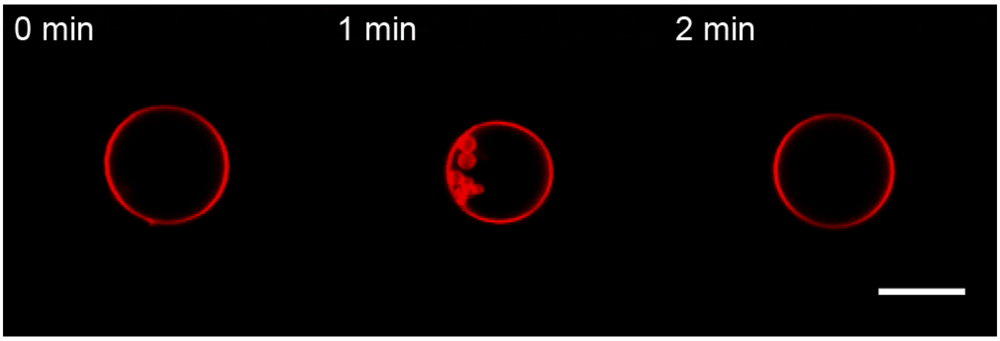

Figure S3. Monitoring the uncharged GUVs from the mixture 1 after 10 seconds of perfusion with the protein-free hyperosmotic solution (1 M NaCl, 50 mM MES, pH 7.0). The scale bar is 10 μm.

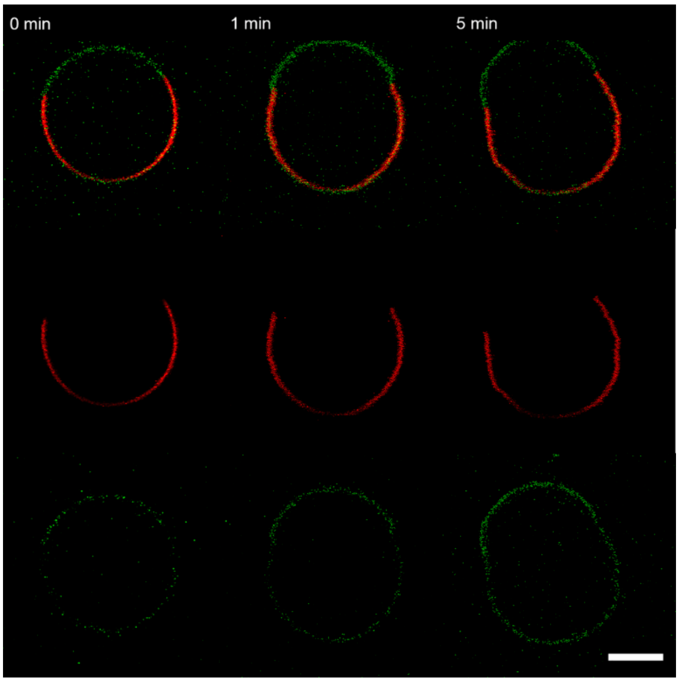

Figure S4. Perfusion with M1 of “raft” GUVs with DOPS instead of bPS (DOPS:Chol:SM:DOPC:BODIPY-GM1:Rho-PE = 20:33.3:33.3:13.3:0.01:0.01 (mol.%)). L_d marker Rho-PE is in the red channel, L_o marker BODIPY-GM1 is in the green channel. The scale bar is 5 μm.

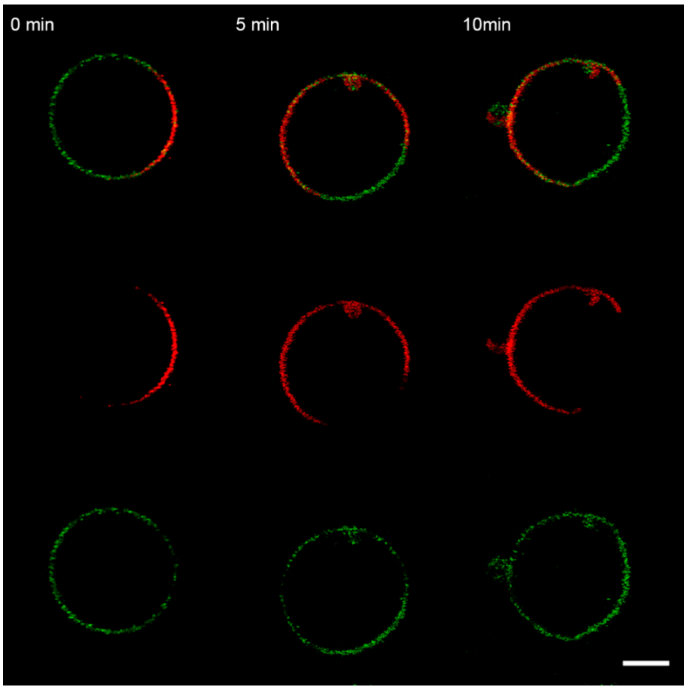

Figure S5. The same as Fig. S4, but for perfusion with BSA. The scale bar is 10 μm.

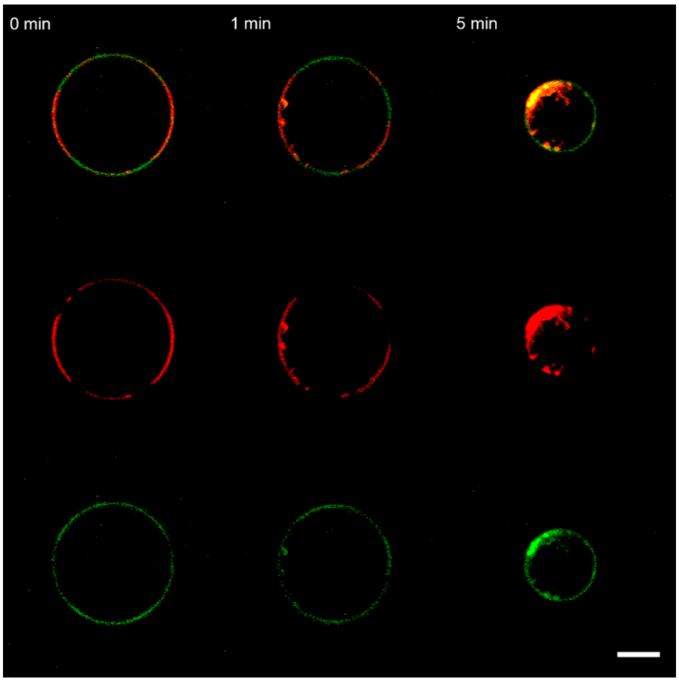

Figure S6. The same as Fig. S4, but for perfusion with PLL. The scale bar is 20 µm.

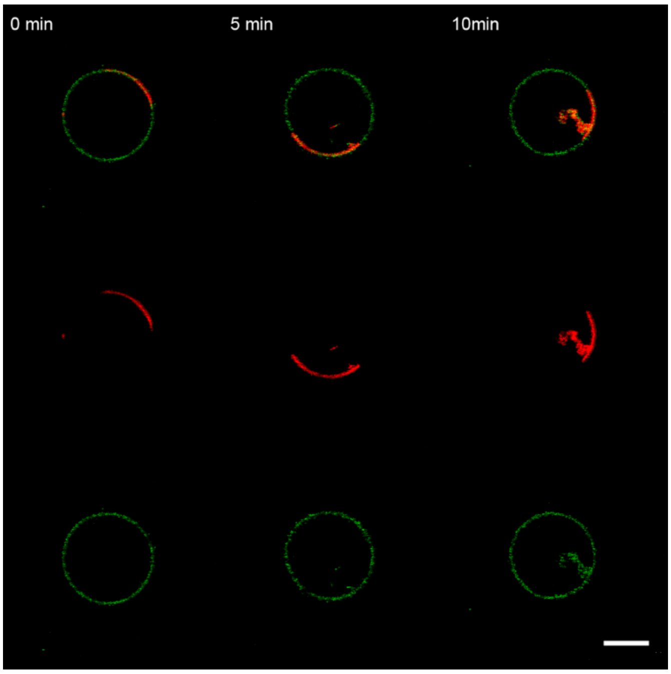

Figure S7. Perfusion with M1 of "raft" GUVs with DSPS instead of bPS (DSPS:Chol:SM:DOPC:BODIPY-GM1:Rho-PE = 20:33.3:33.3:13.3:0.01:0.01 (mol.%)). Color code is the same as in Fig. S4. The scale bar is 10 µm.

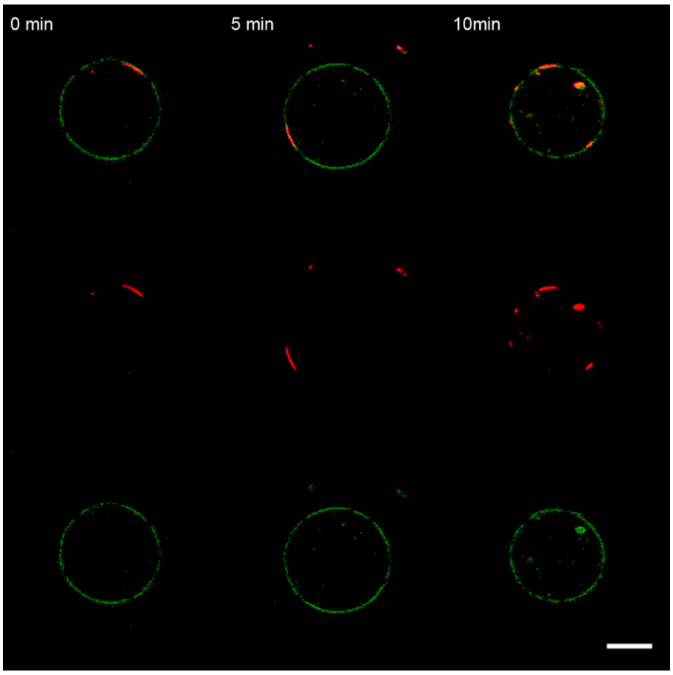

Figure S8. The same as Fig. S7, but for perfusion with PLL. The scale bar is 10 μm.
